# Induction of virus-specific effector immune cell response limits virus replication and severe disease in mice infected with non-lethal West Nile virus Eg101 strain

**DOI:** 10.1186/s12974-015-0400-y

**Published:** 2015-09-22

**Authors:** Mukesh Kumar, Kelsey Roe, Maile O’Connell, Vivek R. Nerurkar

**Affiliations:** Department of Tropical Medicine, Medical Microbiology and Pharmacology, John A. Burns School of Medicine, University of Hawaii at Manoa, 651 Ilalo Street, Honolulu, 96813 Hawaii USA; Pacific Center for Emerging Infectious Diseases Research, John A. Burns School of Medicine, University of Hawaii at Manoa, 651 Ilalo Street, Honolulu, Hawaii 96813 USA

**Keywords:** West Nile virus encephalitis, Neuroinflammation, Immune cell migration

## Abstract

**Background:**

West Nile virus (WNV) is a neurotropic flavivirus that has emerged globally as a significant cause of viral encephalitis in humans. Herein, we investigated the immunological responses induced by two phylogenetically related WNV strains of lineage 1, WNV NY99, and WNV Eg101.

**Methods:**

Eight-week-old C57BL/6J mice were inoculated with WNV NY99 or WNV Eg101 and mortality, virus burden in the periphery and brain, type 1 interferon response, WNV-specific antibodies, leukocyte infiltration, and inflammatory responses were analyzed.

**Results:**

As expected, WNV NY99 infected mice demonstrated high morbidity and mortality, whereas no morbidity and mortality was observed in WNV Eg101 infected mice. Virus titers were comparable in the serum of both WNV NY99 and WNV Eg101 infected mice at day 3 after inoculation; however, at day 6, the virus was cleared from WNV Eg101 infected mice but the virus titer remained high in the WNV NY99 infected mice. Virus was detected in the brains of both WNV NY99 and Eg101 infected mice, albeit significantly higher in the brains of WNV NY99 infected mice. Surprisingly, levels of type 1 interferon and WNV-specific antibodies were significantly higher in the serum and brains of WNV NY99 infected mice. Similarly, protein levels of multiple cytokines and chemokines were significantly higher in the serum and brains of WNV NY99 infected mice. In contrast, we observed significantly higher numbers of innate and adaptive immune cells in the spleens and brains of WNV Eg101 infected mice. Moreover, total number and percentage of IFN-γ and TNF-α producing WNV-specific CD8^+^ T cells were also significantly high in WNV Eg101 infected mice.

**Conclusions:**

Our data demonstrate that induction of virus-specific effector immune cell response limits virus replication and severe WNV disease in Eg101 infected mice. Our data also demonstrate an inverse correlation between leukocyte accumulation and production of pro-inflammatory mediators in WNV-infected mice. Moreover, increased production of pro-inflammatory mediators was associated with high-virus titers and increased mortality in WNV NY99 infected mice.

## Background

West Nile virus (WNV), a neurotropic flavivirus, has emerged as a significant cause of viral encephalitis in the USA [1]. WNV infection in humans is usually asymptomatic or self-limiting, with a mild febrile illness, but may progress to meningitis, encephalitis, paralysis, and death. Until 1999, WNV was geographically distributed in Africa, the Middle East, western and central Asia, India, and Europe, where it caused sporadic cases of febrile disease and occasional outbreaks of encephalitis in elderly people and in equines [2, 3]. The unexpected emergence of WNV in the USA in 1999 was associated with the introduction of the NY99 strain, which is more virulent, and resulted in higher incidence of meningoencephalitis in humans as compared to the non-virulent strains such as the WNV Eg101 strain [4, 5]. Recent outbreaks of highly virulent WNV strains have also been reported in the Mediterranean basin, southern Europe, and Russia [6, 7]. Although the worldwide incidence of WNV infection is increasing, there is no specific treatment or vaccine available for use in humans.

Studies using the well-characterized WNV encephalitis (WNVE) mouse models show that an intact innate and adaptive immune response is required to limit WNV infection. Antiviral type I interferon production is essential in suppressing viral titers in the brain and peripheral organs [8]. The induction of WNV-specific immunoglobulins is essential for suppressing viremia and virus dissemination [9]. In the central nervous system (CNS), neurons are the primary target of WNV replication, and virus-associated pathology is characterized by neuronal death, activation of glial cells, and massive infiltration of leukocytes in the perivascular space and parenchyma. The migration of leukocytes into the brain is essential for controlling WNV infection in the brain [10–12]. WNV also induces a dramatic increase in several pro-inflammatory cytokines such as tumor necrosis factor alpha (TNF-α) and interleukin (IL)-1β and chemokines such as CCl2 and CXCL10, which regulate leukocyte trafficking into the brain and neuronal death after infection [11, 13, 14]. The global increase of WNV neuroinvasive disease warrants a greater understanding of the molecular mechanisms associated with virus clearance and neuroinflammation.

The WNV strain Eg101 was isolated from human blood in Egypt in 1950 [15]. NY99 and Eg101 strains of WNV are 95.4 and 99.6 % identical at the nucleotide and amino acid levels, respectively. Both the WNV NY99 and WNV Eg101 strains are classified in same genotypic lineage and belong to same clade 1a of the lineage 1 [5, 16]. Lineage 1 strains are considered emerging and associated with outbreaks of neuroinvasive disease [5]. While the WNV NY99 strain is highly pathogenic and implicated in large-scale mortality, the WNV Eg101 strain is largely non-pathogenic. We have previously demonstrated that mice immunized with both 1000 and 100 plaque-forming units (PFU) of WNV Eg101 demonstrated 100 % protection against both subcutaneous and intracranial challenge with a lethal dose (1000 PFU) of the WNV NY99 strain [17]. However, the underlying mechanisms associated with differential pathogenesis of lethal, WNV NY99, and non-lethal, WNV Eg101, strains are largely unknown. We hypothesized that differential host immune response is the critical determinant of distinct disease outcome associated with the WNV NY99 and WNV Eg101 strains. In the present study, we examined the immunological changes in the serum and brain of mice during WNV NY99 strain and WNV Eg101 strain infection to understand the underlying immunopathological mechanisms leading to severe WNV disease. While both strains were neuroinvasive in adult mice, our results revealed phenomenal differences in the clinical disease, induction of type 1 interferon and WNV-specific antibodies, innate and adaptive immune cell response, and inflammatory response in the periphery and in the brain.

## Methods

### Ethics statement

This study was specifically approved by the University of Hawaii Institutional Animal Care and Use Committee (IACUC) (protocol number 13–1759), and conducted in strict accordance with guidelines established by the National Institutes of Health and the University of Hawaii IACUC. All animal experiments were conducted in consultation with veterinary and animal care staff at the University of Hawaii in animal biosafety level-3 laboratory, and mice that exhibited severe disease were euthanized to limit suffering. The animal suite was maintained at 72 °F, 45 % humidity, and on 12/12-light/dark cycle. Sawdust bedding was provided along with paper towels.

### WNV infection experiments

Eight-week-old C57BL/6J mice were purchased from the Jackson Laboratory. Mice were inoculated via the footpad route with 100 plaque-forming units (PFU) of Eg101 or NY99 strains of WNV [17, 18]. On specific days after inoculation, 100 μL of blood was collected from the tail vein, and serum was separated and frozen at −80 °C for further analysis. At days 3, 6, 8, and 10 after inoculation, mice were anesthetized using isoflurane and perfused with cold PBS [19, 20]. WNV infection is first detected in the brain between days 4 and 6 after footpad inoculation, and peak virus load is observed at days 8 and 10 after inoculation. Similar to humans, WNV-infected mice demonstrate various neurological symptoms such as paresis, hind limb paralysis, tremors, and ataxic gait [21]. In our model, these symptoms appear at day 8 after inoculation. Mortality is observed between days 8 and 12 after inoculation [19, 20].

Brains were harvested and flash frozen in 2-methylbutane and stored at −80 °C until further processing. Frozen brain tissues were weighed and homogenized in a bullet blender using glass or zirconium oxide beads. WNV titers in the serum and brain homogenates were measured by plaque assay using Vero cells [20, 22]. Alternatively, mice were perfused with PBS followed by 4 % paraformaldehyde (PFA), and brains were harvested, cryoprotected in 30 % sucrose, and embedded in the optimum cutting temperature (OCT) media [23]. In separate experiments, brains and spleens were harvested at day 8 after inoculation for the isolation of leukocytes and were processed for flow cytometry [23].

### Measurement of WNV-specific antibodies

The titers, defined as median fluorescent intensity, of WNV-specific IgM and IgG antibodies were measured in the serum of WNV NY99 and WNV Eg101 infected mice using microsphere immunoassay (MIA) for WNV envelope E protein [17, 24].

### Plaque reduction neutralization test (PRNT)

The titers of anti-WNV neutralizing antibodies were measured in the serum of WNV NY99 and WNV Eg101 infected mice using PRNT assay as described previously [17]. Serum collected from WNV NY99 and WNV Eg101 infected mice were serially diluted from 1:40 to 1:5000, and PRNT was conducted against WNV NY99 and WNV Eg101 viruses, respectively. The highest dilution of serum resulting in 80 % reduction in the number of plaques compared to the growth of the virus control was determined as described previously [17, 24, 25].

### Measurement of cytokines and chemokines

The levels of cytokines and chemokines were measured in the serum and brain homogenates by multiplex immunoassay using MILLIPLEX MAP Mouse Cytokine/Chemokine kit (Millipore) [20, 23].

### ELISA

The levels of IFN-α and IFN-β were measured in the serum and brain homogenates by ELISA, using the VeriKine™ Mouse Interferon-α ELISA Kit and VeriKine™ Mouse Interferon-β ELISA Kit (PBL Interferon Source) [20].

### Immunohistochemistry

Sagittal sections of 10-μm thicknesses were cut from the hemi-brain tissues frozen in OCT, and whole sagittal tissue sections were stained with hematoxylin and eosin (H&E) [23, 26].

### Analysis of splenic leukocytes

Splenocytes were harvested from WNV NY99 and WNV Eg101 infected mice on day 8 after inoculation. Cells were counted and washed with 1X fluorescence-activated cell sorter buffer. Cells were stained for CD45, CD3, NK1.1, CD11b, and Ly6c by directly conjugated antibodies (eBiosciences) for 30 min at 4 °C and then fixed with 2 % PFA. Samples were analyzed by multi-color flow cytometry using FACSAria, and data were analyzed using FlowJo software [19, 23]. Briefly, after gating live cells by forward and side scatter (FSC and SSC) parameters, CD45^+^ leukocytes were selected. CD45^+^ leukocytes were further divided into CD3^+^, CD11b^+^, and NK1.1^+^ cells. CD11b^+^ cells were further delineated into subsets of CD11b^+^ Ly6C^+^ cells. CD3^+^ T cells were further divided into the CD4^+^ and CD8^+^ subsets. The total numbers of CD4^+^ T cells, CD8^+^ T cells, NK1.1^+^ cells, and CD11b^+^ Ly6C^+^ cells from each spleen were determined by multiplying the percentage of these cells by the total number of CD45^+^ leukocytes harvested as described previously [23].

In separate experiments, 10^6^ splenocytes were stimulated with 2 μg/ml of an immunodominant WNV-specific NS4B peptide (SSVWNATTAI) (21^st^ Century Biochemical) for 6 h at 37 °C in the presence of GolgiPlug (BD Biosciences). Splenocytes were stained with anti-CD8 antibody for 30 min at 4 °C followed by fixation and permeabilization (BD Biosciences) and stained with anti-IFNγ and anti-TNF-α antibodies (eBiosciences) for 30 min at 4 °C. Samples were analyzed by multi-color flow cytometry using FACSAria, and the percentages of CD8^+^ T cells that expressed IFNγ or TNF-α were determined using FlowJo software [23].

### Flow cytometric analysis of infiltrated immune cells in the brain

Brains were harvested from WNV NY99 and WNV Eg101 infected mice on day 8 after inoculation and homogenized using a Miltenyi gentle MACS cell dissociator (Miltenyi Biotec) and enzymatic neural dissociation kit (Miltenyi Biotec). Infiltrated leukocytes were isolated by discontinuous Percoll gradient centrifugation. Cells were counted and washed once with PBS. Cells were stained for CD45, CD3, NK1.1, CD11b, and Ly6c by directly conjugated antibodies (eBiosciences) for 30 min at 4 °C and then fixed with 2 % PFA. Samples were analyzed by multi-color flow cytometry using FACSAria, and data were analyzed using FlowJo software [19, 23]. Briefly, after gating live cells by FSC and SSC parameters, CD45^hi^ leukocytes were selected. CD45^hi^ leukocytes were further divided into CD3^+^, CD11b^+^, and NK1.1^+^ cells as described above [23].

### Statistical analysis

Log-rank (Mantel-Cox) Test and Gehan-Breslow-Wilcoxon Test were used to analyze survival data. Mann–Whitney test and unpaired Student *t* test using GraphPad Prism 5.0 was used to calculate *p* values of difference between viral titers and immune responses, respectively. Differences of *p* < 0.05 were considered significant.

## Results

### Morbidity and mortality in mice following WNV NY99 and WNV Eg101 infection

**Fig. 1:**
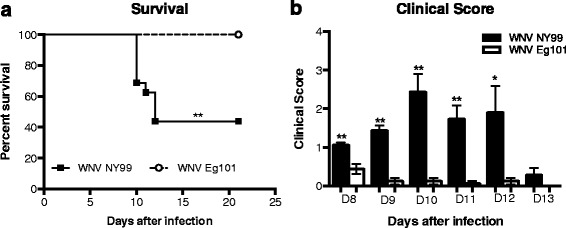
Survival analysis and clinical score of WNV NY99 and WNV Eg101 infected mice. **a** Eight-week-old C57BL/6J mice were inoculated subcutaneously with 100 PFU of WNV NY99 or WNV Eg101. All mice were observed for 21 days. Data are combined of three independent studies (*n* = 24 mice per group). **b** Animals were monitored for clinical scores twice a day. The designation for the clinical scores is as follows: 1, ruffled fur/hunched back; 2, paresis/difficulty walking; 3, paralysis; 4, moribund/euthanized; and 5, dead. *Error bars* represent SEM. *p < 0.001, **p < 0.0001

Eight-week-old C57BL/6J mice were inoculated subcutaneously with 100 PFU of WNV Eg101 or WNV NY99 or PBS (mock) and then monitored daily for clinical signs and mortality. PBS inoculated mice remained healthy throughout the observation period of 21 days. WNV NY99 inoculated mice began to demonstrate clinical signs at day 8 after inoculation and exhibited high (65 %) mortality. In comparison, Eg101 inoculated mice rarely displayed clinical signs of disease and no mortality was observed in these mice (Fig. 1a, b). The difference in mortality between WNV NY99 and WNV Eg101 infected mice was statistically significant. All surviving animals were positive for anti-WNV IgG antibodies, confirming virus infection (data not shown).

### Viral load in the serum and brains of WNV NY99 and WNV Eg101 infected mice

**Fig. 2:**
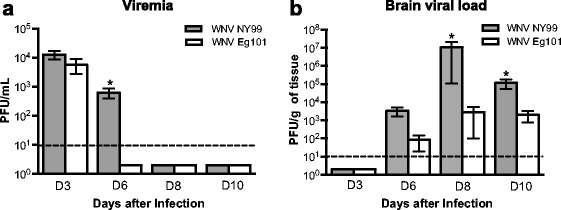
Virus titers in the serum and brains of WNV NY99 and WNV Eg101 infected mice. The kinetics of virus replication and levels of WNV were determined in the serum (**a**) and brains (**b**) of WNV NY99 or WNV Eg101 infected mice at indicated time points (days 3, 6, 8, and 10) by plaque assay. The data are expressed as PFU/mL of serum and PFU/g of brain tissue. *Error bars* represent SEM (*n* = 6–10 mice per group). *p < 0.05. *Horizontal dotted line* indicates the limit of detection

We first examined the WNV titers in the serum and brains of WNV NY99 and WNV Eg101 infected mice by plaque assay. High, but similar virus titers were detected by plaque assay in the serum of both WNV NY99 and WNV Eg101 infected mice at day 3 after inoculation (Fig. 2a). At day 6 after inoculation, virus was cleared from the periphery of all the WNV Eg101 infected mice; however, it remained significantly high in WNV NY99 infected mice. The virus was cleared from the periphery of all WNV NY99 infected mice by day 8 after inoculation. WNV was not detected in the brains of both WNV NY99 and WNV Eg101 infected mice at day 3 after inoculation, which is consistent with the previous studies [20]. WNV was first detected in the brains at day 6 after inoculation and peaked at day 8 after inoculation (Fig. 2b). WNV titers in the brains of WNV NY99 infected mice was slightly higher than WNV Eg101 infected mice at day 6 after inoculation, but the difference was not statistically significant. However, virus titers were significantly higher in the brains of WNV NY99 infected mice when compared to WNV Eg101 infected mice at both days 8 and 10 after inoculation.

### WNV-specific antibodies and type 1 interferon responses in mice following WNV NY99 and WNV Eg101 infection

**Fig. 3:**
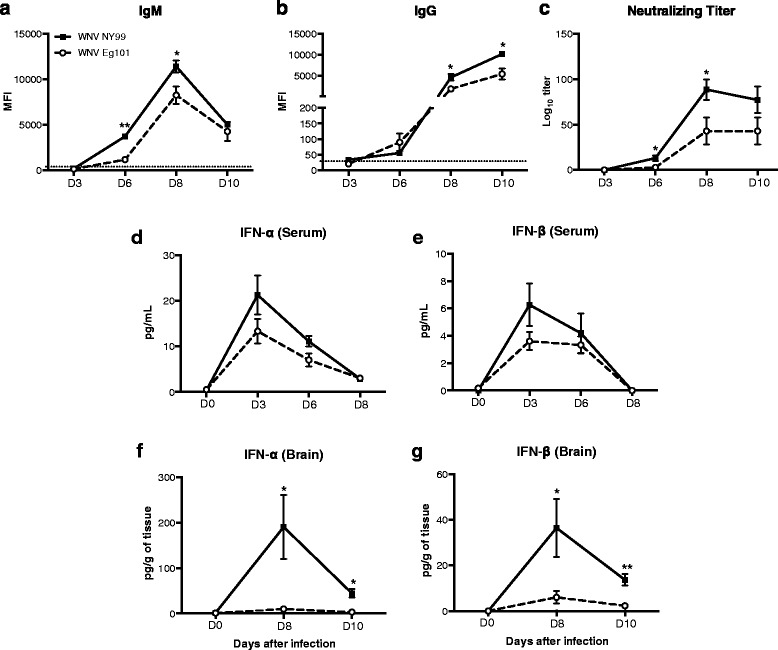
WNV-specific IgM and IgG titers, neutralizing antibodies titers, and levels of IFN-α and IFN-β in mice following WNV NY99 and WNV Eg101 infection. Serum was collected from WNV NY99 and WNV Eg101 infected mice at indicated time points and WNV-specific IgM (**a**) and IgG (**b**) responses were measured by MIA using WNV E antigen. Results are reported as median fluorescent intensity (MFI) per 100 microspheres. Data are expressed as MFI ± SEM and is representative of two independent experiments (*n* = 8–16 mice per group). *p < 0.05, **p < 0.001. *Horizontal dotted line* indicates the cutoff value. **c** Neutralizing antibodies titers in the serum of WNV NY99 and WNV Eg101 infected mice. Serum collected from WNV NY99 and WNV Eg101 infected mice at indicated time points was serially diluted from 1:40 to 1:5000 and PRNT was conducted against WNV NY99 and WNV Eg101 viruses, respectively. Data are expressed as mean log_10_ titer ± SEM and is representative of two independent experiments (*n* = 8–16 mice per group). IFN-α and IFN-β production was measured in the serum (**d**) and (**e**) collected from WNV NY99 and WNV Eg101 infected mice at indicated time points using mouse IFN-α and mouse IFN-β ELISA kits and from brains (**f**) and (**g**) harvested from WNV NY99 and WNV Eg101 infected mice at days 6 and 8 after inoculation. The data are expressed as pg/mL of serum or pg/g of brain tissue. *Error bars* represent SEM (*n* = 6–8 mice per group). *p < 0.05, **p < 0.001

The titers of WNV-specific IgM and IgG antibodies in the serum of WNV NY99 and WNV Eg101 infected mice were measured using MIA. Infection with both WNV NY99 and WNV Eg101 elicited robust WNV-specific antibody responses. Anti-WNV IgM first appeared at day 6 peaked at day 8 and then decreased at day 10 after inoculation (Fig. 3a). Similarly, anti-WNV IgG first appeared at day 6 and gradually increased up to day 10 after inoculation (Fig. 3b). However, titers of both IgM (days 6 and 8) and IgG (days 8 and 10) antibodies were significantly higher in WNV NY99 infected mice than WNV Eg101 infected mice. Similarly, titers of neutralizing antibodies were also significantly higher in WNV NY99 infected mice than WNV Eg101 infected mice (Fig. 3c).

We measured the levels of IFN-α and IFN-β in the serum of WNV NY99 and WNV Eg101 infected mice using ELISA. High levels of IFN-α and IFN-β were detected in the serum of both WNV NY99 and WNV Eg101 infected mice at day 3 after inoculation, which decreased gradually at days 6 and 8 after inoculation. However, as demonstrated in Fig. 3d, e, we observed a trend of slightly reduced levels of IFN-α and IFN-β in the serum of WNV Eg101 infected mice at days 3 and 6 after inoculation. We also examined IFN-α and IFN-β levels in the brains at days 8 and 10 after inoculation. In the brain, as compared to WNV Eg101 which did not elicit a strong interferon response, WNV NY99 infected mice developed a robust interferon response at day 8 after inoculation. This response further decreased by day 10 after inoculation (Fig. 3f, g).

### Peripheral cytokine and chemokine profiles in mice after WNV NY99 and WNV Eg101 infection

**Fig. 4:**
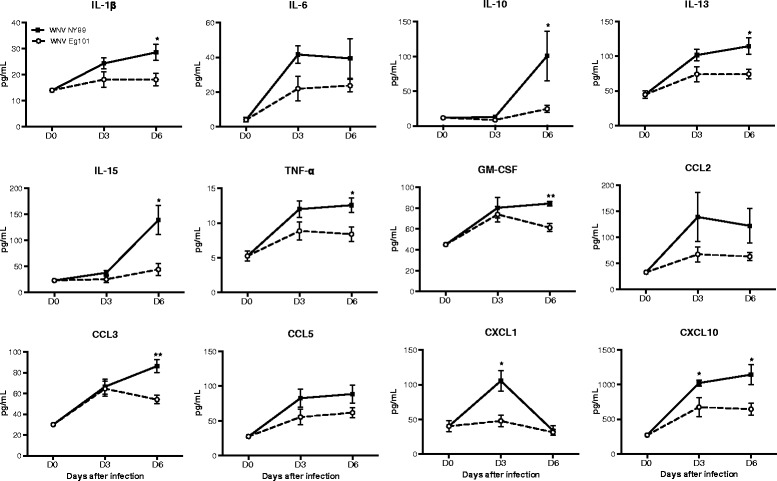
Cytokines and chemokines levels in the serum of WNV NY99 and WNV Eg101 infected mice. Serum was collected from WNV NY99 and WNV Eg101 infected mice at indicated days after virus inoculation. Levels of chemokines and cytokines as noted in the figure were measured using multiplex immunoassay and are expressed as the mean concentration (pg/mL) ± SEM, representing two independent experiments (*n* = 6–10 mice per group). *p < 0.05, **p < 0.001

It has been demonstrated that WNV-induced expression of chemokines such as CCL2, CCL3, CCL4, CCL5, and CXCL10 are associated with enhanced trafficking of leukocytes into the brain [11, 27–29, 30–32]. WNV-induced expression of pro-inflammatory cytokines such as IL-1β and TNF modulate BBB permeability, facilitate leukocyte trafficking into the brain, activate glial cells, and mediate neuronal death after infection [13, 14]. Therefore, we next assessed the protein levels of these cytokines and chemokines in the serum of WNV NY99 and WNV Eg101 infected mice using multiplex immunoassay. Very low levels of these cytokines and chemokines were detected in the serum of uninfected mice (day 0). WNV NY99 infection resulted in a dramatic increase in protein levels of multiple cytokines and chemokines at day 3 after inoculation, which further increased slightly at day 6 after inoculation (Fig. 4). We also observed a slight increase in the levels of these cytokines and chemokines in WNV Eg101 infected mice at day 3 after inoculation. However, unlike WNV NY99 infected mice, there was no increase in their levels at day 6 after inoculation. Levels of interleukin (IL)-1β, −10, −13, and −15 were significantly higher in WNV NY99 infected mice than WNV Eg101 infected mice at day 6 after inoculation. Similarly, TNF-α and GM-CSF levels in the serum of WNV NY99 infected mice were significantly higher when compared to WNV Eg101 infected mice at day 6 after inoculation. A similar pattern was also observed for chemokine expression. CCL3 level was significantly higher in WNV NY99 infected mice at day 6 after inoculation, whereas the level of CXCL10 was significantly higher in WNV NY99 infected mice at both days 3 and 6 after inoculation. The level of CXCL1 was only increased significantly at day 3 after inoculation when compared to WNV Eg101 mice. However, the comparison of levels of serum cytokines and chemokines, such as IL-6, CCL2, and CCL5 between both groups demonstrated no significant differences in their levels at any time point after inoculation (Fig. 4).

### Cytokine and chemokine profiles in the brains of WNV NY99 and WNV Eg101 infected mice

**Fig. 5:**
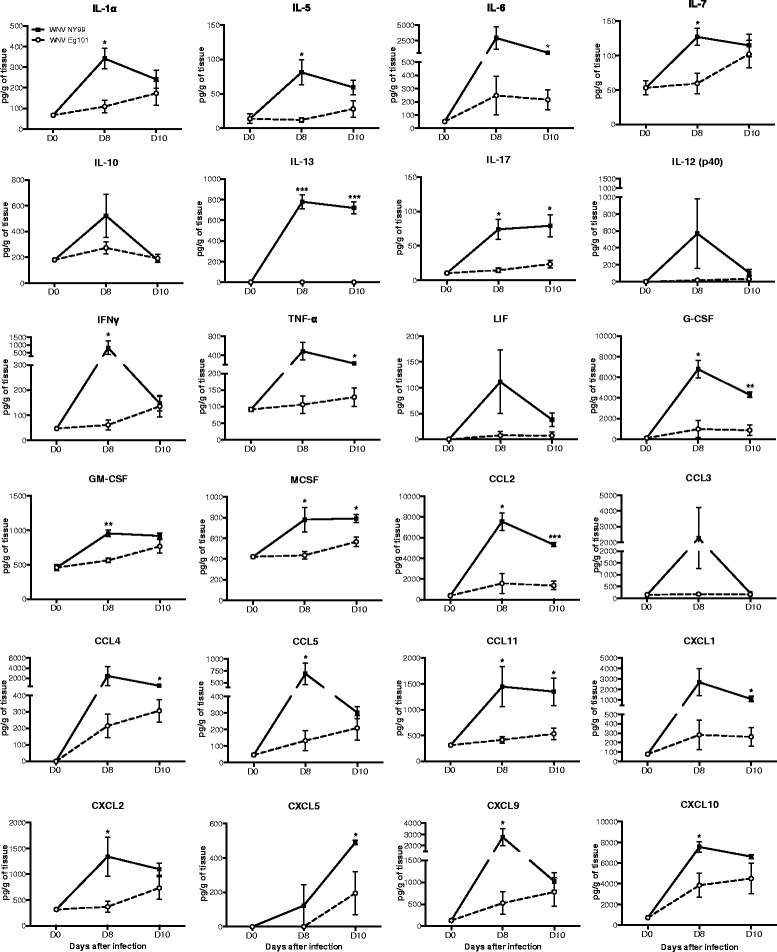
Cytokines and chemokines levels in the brains of WNV NY99 and WNV Eg101 infected mice. Brains were harvested from WNV NY99 and WNV Eg101 infected mice at indicated time points and homogenized as described in the materials and methods section. Levels of cytokines and chemokines as noted in the figure were measured using multiplex immunoassay and are expressed as the mean concentration (pg/g of tissues) ± SEM, representing two independent experiments (*n* = 6 mice per group). *p < 0.05, **p < 0.001, ***p < 0.0001

We further examined the protein levels of multiple inflammatory cytokines and chemokines in the mice brain homogenates following WNV NY99 and WNV Eg101 infection. Very low levels of these cytokines and chemokines were detected in the brains of uninfected mice (day 0). As expected, levels of multiple cytokines and chemokines increased dramatically in WNV NY99 infected mice brains at day 8 after inoculation, but this increase was not observed in the brains of WNV Eg101 infected mice (Fig. 5). Levels of these cytokines and chemokines decreased slightly in the brains of WNV NY99 infected mice at day 10 after inoculation. In contrast, there was a slight increase in the levels of these inflammatory mediators in the brains of WNV Eg101 infected mice at day 10 after inoculation. The levels of IL-1α, IL-5, IL-7, IFNγ, GM-CSF, CCL5, CXCL2, and CXCL9 were significantly higher in the brains of WNV NY99 infected mice than WNV Eg101 infected mice at day 8 after inoculation. Similarly, the levels of IL-13, IL-17, G-CSF, MCSF, CCL2, and CCL11 were significantly higher in WNV NY99 infected mice at both days 8 and 10 after inoculation when compared to WNV Eg101 mice. However, the levels of IL-6, TNF-α, CCL4, CXCL1, and CXCL5 were increased significantly only at day 10 after inoculation when compared to WNV Eg101 mice. Whereas, levels of IL-10, IL-12, LIF, and CCL3 did not differ between WNV NY99 and WNV Eg101 infected mice at any time point after inoculation (Fig. 5).

### Leukocyte accumulation in the spleens of WNV NY99 and WNV Eg101 infected mice

**Fig. 6:**
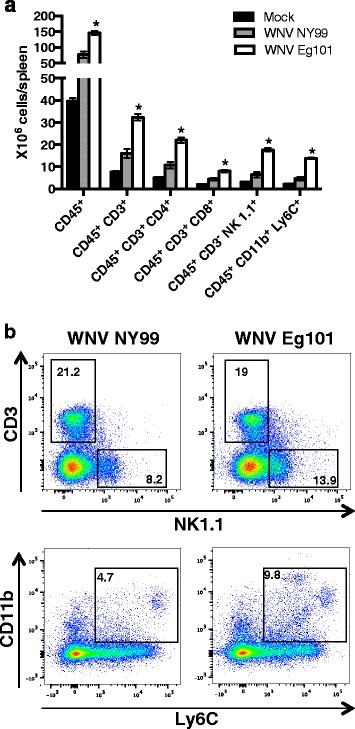
Leukocyte accumulation in the spleens of WNV NY99 and WNV Eg101 infected mice. Spleens were harvested from mock, WNV NY99, and WNV Eg101 infected mice after extensive perfusion at day 8 after inoculation. Splenocytes were isolated and analyzed by flow cytometry. **a** Total number of leukocytes (CD45^+^), T cells (CD45^+^ CD3^+^), CD4^+^ T cells (CD45^+^ CD3^+^ CD4^+^), CD8^+^ T cells (CD45^+^ CD3^+^ CD8^+^), NK cells (CD45^+^ CD3^−^ NK1.1^+^), and monocytes/macrophages (CD45^+^ CD11b^+^ Ly6c^+^) per spleen. Data represents the mean ± SEM, representing two independent experiments (*n* = 6 mice per group). *p < 0.05. **b** Representative flow cytometry profiles demonstrating percentages of NK cells, and monocytes/macrophages in the spleen at day 8 after inoculation. Data represents two independent experiments (*n* = 6 mice per group)

Splenocytes were isolated from mock, WNV NY99 and WNV Eg101 infected mice at day 8 after inoculation and were analyzed by flow cytometry (Fig. 6). Cells were stained for CD45, CD3, CD4, CD8, NK1.1, CD11b, and Ly6C markers to analyze both T cells and non-T cell subpopulations. Interestingly, WNV Eg101 infected mice spleens had significantly higher numbers of total leukocytes (CD45^+^ cells), CD4^+^ T cells (CD45^+^ CD3^+^ CD4^+^), CD8^+^ T cells (CD45^+^ CD3^+^ CD8^+^), NK cells (CD45^+^ CD3^−^ NK1.1^+^), and monocytes/macrophages (CD45^+^ CD11b^+^ Ly6c^+^) than WNV NY99 infected mice spleen (Fig. 6a). Additionally, the percentage of NK cells and monocytes/macrophages in the spleens of WNV Eg101 infected mice were also higher than in WNV NY99 infected mice (Fig. 6b). No difference was observed in the percentage of T cells in the spleens of WNV Eg101 and NY99 infected mice.

### WNV-specific T cell responses in WNV NY99 and WNV Eg101 infected mice

**Fig. 7:**
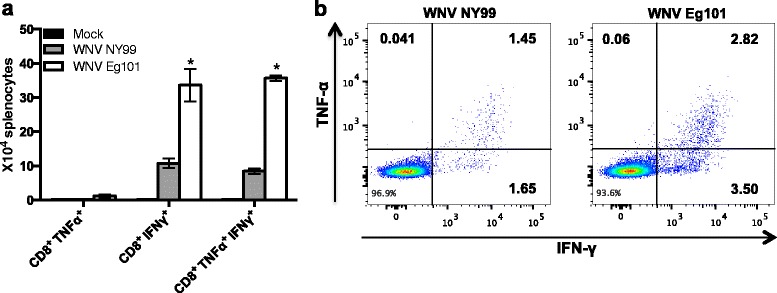
T cell responses in the spleens following WNV NY99 and WNV Eg101 infection. Spleens were harvested after extensive perfusion of mock, WNV NY99, and WNV Eg101 infected mice at day 8 after inoculation. Splenocytes were isolated and stimulated ex vivo for 6 h with an immunodominant NS4B peptide as described in the materials and methods. Cells were co-stained for CD8 and intracellular IFN-γ and TNF-α and analyzed by flow cytometry. **a** Total number of CD8^+^ TNF-α^+^ cells, CD8^+^ IFNγ^+^ cells, and CD8^+^ TNF-α^+^ IFNγ^+^ cells. Data represents the mean ± SEM, representing two independent experiments (*n* = 6 mice per group) *p < 0.05, ***p < 0.0001. **b** Representative flow cytometry profiles demonstrating percentages of CD8^+^ TNF-α^+^ cells, CD8^+^ IFNγ^+^ cells, and CD8^+^ TNF-α^+^ IFNγ^+^ cells. Data represents two independent experiments (*n* = 6 mice per group)

Splenocytes from mock, WNV NY99, and WNV Eg101 infected mice were harvested at day 8 after inoculation and re-stimulated ex vivo with an immunodominant WNV NS4B peptide, and CD8^+^T cells were analyzed for the production of intracellular IFNγ and TNF-α by flow cytometry. As depicted in Fig. 7a, b, total number and percentage of CD8^+^ IFNγ^+^ cells (3.50 versus 1.65 %) and CD8^+^ IFNγ^+^ TNF-α^+^ cells (2.82 versus 1.45 %) were significantly higher in WNV Eg101 infected mice than in WNV NY99 infected mice, respectively. We observed no significant difference in the percentage of CD8^+^ TNF-α^+^ cells from WNV NY99 and WNV Eg101 infected mice after incubation with WNV-specific peptide.

### Leukocyte infiltration in the brain after WNV NY99 and WNV Eg101 infection

**Fig. 8:**
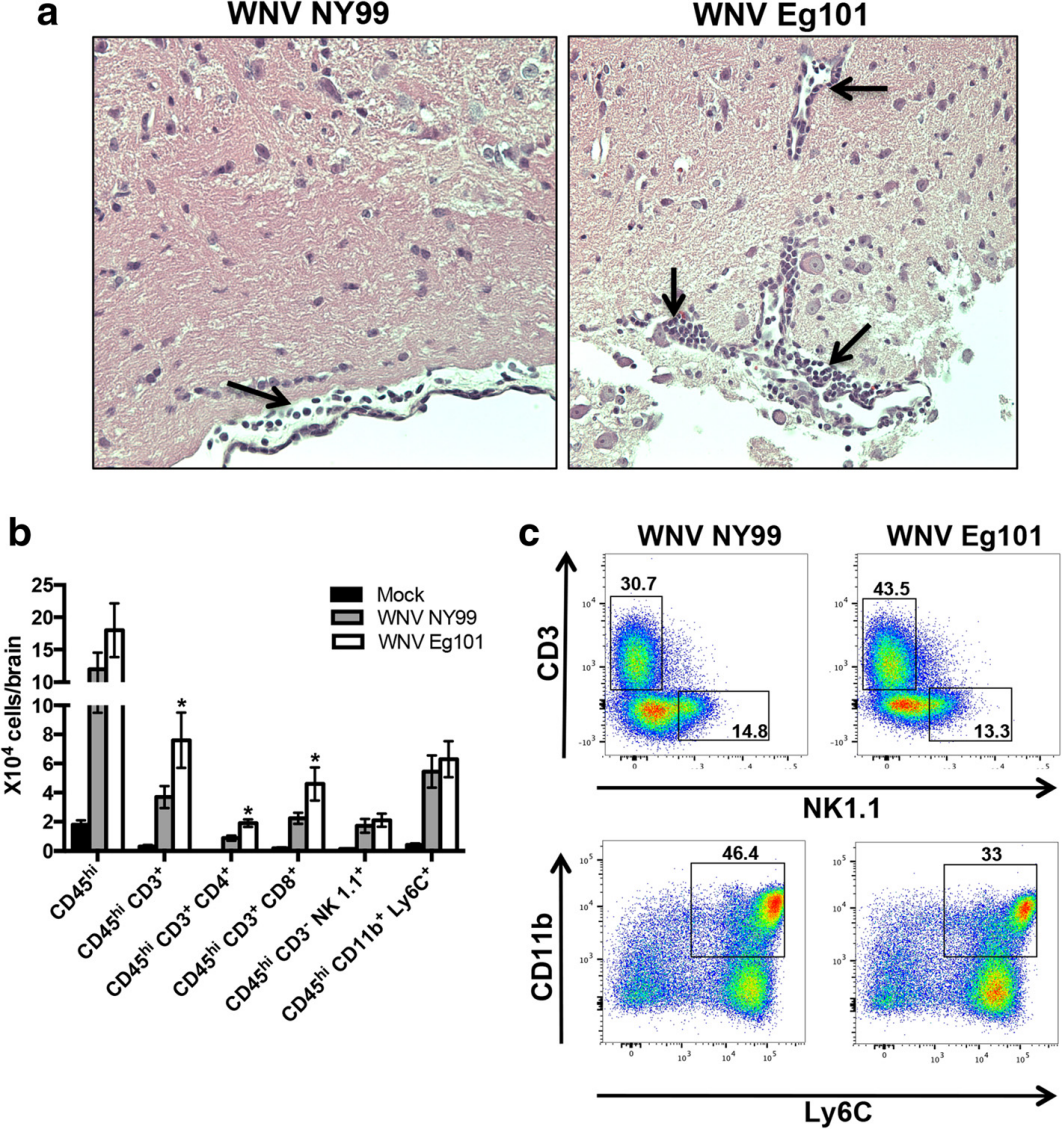
Infiltration of leukocytes in the brains of WNV NY99 and WNV Eg101 infected mice. **a** PFA-fixed brain sections from WNV NY99 and WNV Eg101 infected mice at day 8 after inoculation were stained with hematoxylin and eosin (H & E). *Black arrows* identify leukocytes infiltrating into the meninges. The photomicrographs demonstrate representative images obtained from two independent experiments (*n* = 4 mice per group). **b** Brains were harvested from mock, WNV NY99, and WNV Eg101 infected mice at day 8 after inoculation, and leukocytes were isolated by Percoll gradient centrifugation. Total number of leukocytes (CD45^hi^), T cells (CD45^hi^ CD3^+^), CD4^+^ T cells (CD45^hi^ CD3^+^ CD4^+^), CD8^+^ T cells (CD45^hi^ CD3^+^ CD8^+^), NK cells (CD45^hi^ CD3^−^ NK1.1^+^), and monocytes/macrophages (CD45^hi^ CD11b^+^ Ly6c^+^) per brain were analyzed using flow cytometry. Data represents the mean ± SEM, representing two independent experiments (*n* = 6 mice per group). *p < 0.05. **c** Representative flow cytometry profiles demonstrating percentages of T cells and monocytes/macrophages in the brains at day 8 after inoculation. Data represents two independent experiments (*n* = 6 mice per group)

We initially examined the mice brain tissues for histopathological changes following WNV NY99 and WNV Eg101 infection. As expected, H&E staining of brain sections from the WNV NY99 infected mice demonstrated leukocyte infiltration along the meninges at day 8 after inoculation (Fig. 8a). Interestingly, we observed higher numbers of leukocytes in the meninges and parenchyma of WNV Eg101 infected mice. To further verify these results, we isolated leukocytes from the brains of mock, WNV NY99, and WNV Eg101 infected mice at day 8 after inoculation and analyzed by flow cytometry. Consistent with our immunohistochemistry results, we observed higher numbers of total leukocytes (CD45^hi^ cells) in the brains of WNV Eg101 infected mice (Fig. 8b), which was statistically not significant. However, the total number of CD4^+^ T cells (CD45^hi^ CD3^+^ CD4^+^) and CD8^+^ T cells (CD45^hi^ CD3^+^ CD8^+^) trafficking into the brains of WNV Eg101 infected mice were significantly higher than in WNV NY99 infected mice. There was no difference between the numbers of NK cells (CD45^hi^ CD3^−^ NK1.1^+^) and monocytes/macrophages (CD45^hi^ CD11b^+^ Ly6c^+^) in the brains of WNV Eg101 and WNV NY99 infected mice. As demonstrated in Fig. 8c, the percentage of T cells in the brains of WNV Eg101 infected mice was higher than in those of WNV NY99 infected mice (43.5 versus 30.7 %). On the other hand, unlike the spleen, the percentage of monocytes/macrophages was lower in the brains of WNV Eg101 infected mice (33 versus 46.4 %). No difference between percentage of NK cells in the brains of WNV Eg101 and WNV NY99 infected mice was observed.

## Discussion

In this study, NY99 and Eg101, two phylogenetically closely related lineage 1 WNV strains (95.4 and 99.6 % identical at the nucleotide and amino acid level, respectively) of contrasting pathogenicity, were compared with regard to induction of clinical disease and immune responses in the periphery and brain of mice. We observed that both viral strains were neuroinvasive and induced a robust anti-WNV immune response when inoculated subcutaneously at similar doses. While the production of type 1 interferon and WNV-specific antibodies were higher in WNV NY99 infected mice, the innate and adaptive cellular immune response was more predominant in WNV Eg101 infected mice. Furthermore, by demonstrating that immune cells accumulation was higher in the spleens and brains of mice infected with the non-virulent WNV strain, we suggest that pathogenicity is not directly correlated to the number of immune cells in the spleen and brain. Moreover, our results demonstrating an inverse correlation between the number of immune cells in the brain and the levels of pro-inflammatory mediators indicate that WNV-infected/activated resident brain cells not infiltrating immune cells are the primary source of these inflammatory mediators and their increased levels correlate with high brain viral load and severe WNV disease.

### Enhanced WNV-specific antibodies, type 1 interferon and inflammatory response in WNV NY99 infected mice

Despite the high degree of homology between the two strains, WNV Eg101 is non-pathogenic in adult mice after peripheral inoculation. WNV Eg101 infected mice rarely displayed clinical signs and no mortality was observed in these mice [17]. Similar to our study, it has been demonstrated that peripheral inoculation of the Eg101 strain into MBR/ICR albino Swiss mice rarely resulted in fatal illness in mice older than 4 weeks. However, direct intracranial inoculation of WNV Eg101 in same age-matched mice was lethal [33], thereby suggesting the role of peripheral immune response in limiting virus replication, neuroinvasion, and severe WNV disease in WNV Eg101-infected mice. Interestingly, initial virus replication was similar in mice after infection with both WNV NY99 and WNV Eg101 strains as demonstrated by high and comparable viremia at day 3 after inoculation in both the groups. However, viremia in WNV Eg101 infected mice was short-lived than WNV NY99 infected mice, suggesting the induction of an early and effective immune response in these mice. It has been demonstrated that type I interferon and WNV-specific immunoglobulins are rapidly produced following WNV infection and are essential for suppressing viremia and virus dissemination [8, 9]. In this study, we also observed a rapid and robust production of type I interferon and WNV-specific antibodies in both groups. Surprisingly, we observed reduced levels of IFN-α and IFN-β, WNV-specific IgM and IgG antibodies and neutralizing antibodies, in the serum of WNV Eg101 infected mice than WNV NY99 infected mice.

Similar to interferon and WNV-specific immunoglobulins, WNV-induced pro-inflammatory mediators are also known to protect mice from lethal WNV disease [11, 14, 31]. TNF-α and IL-1β promotes immune cell trafficking into the brain [14, 31]. CXCL10 promotes trafficking of WNV-specific CD8^+^ T cells via binding to its cognate receptor CXCR3 [11]. Enhanced expression of CCL3, CCL4, and CCL5 by WNV infection leads to CCR5-dependent trafficking of CD4^+^ and CD8^+^ T cells, NK cells, and macrophages into the brain [32]. CCL2 is important in the trafficking of inflammatory monocyte into the brain after WNV infection [33]. Interestingly in this study, we observed an enhanced production of key pro-inflammatory cytokines and chemokines in the serum of WNV NY99 infected mice than WNV Eg101 infected mice despite increased mortality observed in WNV NY99 infected mice. One possible explanation for these differences is increased viral antigen load in WNV NY99 infected mice, leading to enhanced type 1 interferon, antibodies, and inflammatory mediators in these mice.

### Increased WNV-specific innate and adaptive immune cell response in WNV Eg101 infected mice

WNV dissemination is also countered by the effector functions of innate and adaptive immune cells. Several studies have demonstrated that immune cells such as monocyte/macrophages and T cells play an indispensable role in the control of WNV infection [10, 12, 34]. The role of NK cells in regulating tissue tropism to WNV infection or in controlling WNV replication in vivo has not been clearly defined [35, 36]. However, various in vitro and ex vivo studies have suggested their role in the control of WNV infection by their recognition and elimination of infected cells [37, 38].

Unlike the interferon and inflammatory response, we observed significantly higher numbers of total leukocytes, T cells, NK cells, and monocytes/macrophages in the spleens of WNV Eg101 infected mice. Moreover, total number and percentage of IFN-γ and TNF-α producing WNV-specific CD8 T cells were also significantly higher in WNV Eg101 infected mice. This is surprising as interferon and various inflammatory mediators have been demonstrated to enhance immune cell responses in WNV infection [39–41]. Another interesting finding is the increased percentage of NK cells and monocytes/macrophages observed in the spleens of WNV Eg101 infected mice suggesting a predominant role of innate immune cells in limiting virus replication in these mice. Collectively, these data demonstrate that induction of virus-specific effector immune cell response limits virus replication and severe WNV disease in Eg101 infected mice.

It could be argued that differences in virulence observed after infection with WNV NY99 and WNV Eg101 strains in this study are virus dose-dependent. For example, a lower dose (10 PFU) of WNV NY99 could give similar results to those seen with 100 PFU of WNV Eg101, or a higher dose of WNV Eg101 (1000 PFU) could give similar results to those seen with 100 PFU of WNV NY99. However, our previously published data demonstrate that a lower dose (10 PFU) of WNV NY99 results in 37 % mortality when compared to 0 % mortality as observed with 100 PFU of WNV Eg101 [19, 20]. This data clearly demonstrates that a lower dose of WNV NY99 (10 PFU) does not give similar results to those observed with 100 PFU of WNV Eg101. Moreover, our published data demonstrate no significant difference in terms of survival, clinical score, viremia, WNV-specific IgG and IgM antibodies, and neutralizing antibodies in mice infected with different doses (100 or 1000 PFU) of WNV Eg101 [17].

WNV entry and replication into the CNS represents an important event in the clinical outcome of WNV disease. Following WNV infection, immune cells, especially T cells and macrophages, travel into the brain and participate in viral clearance [10, 12, 34]. Moreover, the production of IFN-α and –β is essential in suppressing virus replication in the brain [8]. In this study, we observed significantly higher virus titers in the brains of WNV NY99 infected mice when compared to WNV Eg101 infected mice at both days 8 and 10 after inoculation. Similar to the periphery, the levels of IFN-α and -β were significantly lower in the brains of WNV Eg101 infected mice. Interestingly, similar to the spleen, the total number of immune cells migrating into the brains of WNV Eg101 infected mice was higher than in WNV NY99 infected mice. However, unlike the spleen, we only observed a significantly higher number of T cells in the brains of WNV Eg101 infected mice. No difference was observed between the number of NK cells and monocytes/macrophages in the brains of WNV Eg101 and WNV NY99 infected mice. Similarly, we also observed a high percentage of T cells in the brains of WNV Eg101 infected mice. On the other hand, we observed a low percentage of monocytes/macrophages in the brains of WNV Eg101 infected mice. Collectively, these data are consistent with previous observations that virus-specific T cells are the predominant cell types in clearing WNV from the brain.

Absence of functional CD8^+^ or CD4^+^ T cells resulted in failure to clear WNV from infected neurons in the CNS [10,12]. It has been demonstrated that increased CD8^+^ T cells contribute to the immunopathology with high doses of WNV challenge [42]. It is also known that migration of inflammatory monocytes plays a pathogenic role during WNVE [33, 43, 44]. Similarly, immune cell-mediated pathology in the brain has been demonstrated in several neurotropic virus infections such as tick-borne encephalitis virus, Murray Valley encephalitis virus, and Theiler's murine encephalomyelitis virus [45–48]. However, in this study, we did not observe any immunopathology associated with increased immune cell migration into the brains of WNV Eg101 infected mice. This may be due to significantly low levels of inflammatory mediators in the brains of WNV Eg101 infected mice.

### Negative correlation between migrating immune cells and associated inflammation

One of the most intriguing findings of our study was the difference in the inflammatory responses observed in the brain. The migration of leukocytes into WNV-infected brain is associated with increased expression of several cytokines and chemokines in the brain. Also, increased expression of cytokines and chemokines in the WNV-infected brain is usually associated with enhanced trafficking of leukocytes into the brain [11, 14, 32]. In contrast, we demonstrate that increased accumulation of leukocytes in the brains of WNV Eg101 (non-virulent strain) infected mice was associated with reduced production of pro-inflammatory cytokines and chemokines. This phenomenon of significantly increased migration of leukocytes in to the brain of WNV Eg101 infected mice despite reduced production of chemokines and cytokines could be due to the higher numbers of WNV-specific primed cells in the periphery of these mice. Lower numbers of infiltrating cells in the brains of WNV NY99 infected mice could be due to different chemokine receptor expression on the leukocytes leading to differential responsiveness to the chemokines emanating from the brain. Differential virus cytopathic effects of WNV NY99 and WNV Eg101 viruses could also result in difference in leukocytes numbers observed in the brain and spleen. Moreover, we observed a dramatic increase in various chemokine and cytokine levels in the brains of WNV NY99 (virulent strain) mice, which correlates with high brain viral load detected in these mice. These data indicate that infiltrating immune cells may play a limited role in the production of these inflammatory mediators and WNV-infected/activated resident brain cells are the primary source(s) of these inflammatory mediators in the brain. However, our data do not rule out the possibility that monocytes/macrophages/microglial may be less activated in Eg101 infected mice brains and may produce less pro-inflammatory cytokines, or the infiltrating immune cell population is suppressing/regulating brain cytokine production.

Resident brain cells such as neurons and astrocytes have been associated with producing inflammatory mediators in several neuroinflammatory scenarios including WNV infection. It has been previously demonstrated by us and others that WNV-infected neurons directly contribute to inflammation by secreting various chemokines and cytokines [11, 13]. Similarly, we and others have previously demonstrated that WNV-infected and activated astrocytes can produce high levels of cytokines and chemokines [49, 50]. Recent studies of WNV infection in brain slices cultures demonstrate the ability of the brain cells to produce pro-inflammatory cytokine response and to activate astrocytes and microglial cells in the absence of infiltrating inflammatory cells and systemic immune responses [51, 52].

Neuronal death is the hallmark of WNVE. Like several encephalitic viruses, WNV induces neuronal death both in a direct or indirect manner, and in the latter case, through inflammation [13, 53, 54]. Therefore, increased neuronal death and subsequent pathology in WNV NY99 infected mice could be due to (i) virus-mediated neuronal apoptosis and (ii) bystander neuronal damage due to pro-inflammatory molecules released by resident brain cells, such as neurons and activated glial cells. Taken together, these data demonstrate that induction of effective and balanced immune response in the periphery and brain of WNV Eg101 infected mice limits virus replication and severe WNV disease in these mice.

## Conclusions

In conclusion, we demonstrate that effector functions of specific innate and adaptive immune cell subsets protect mice against WNV Eg101 disease. Increased migration of immune cells in the brain is not associated with enhanced production of pro-inflammatory mediators and associated immunopathology in mice. In contrast, increased production of pro-inflammatory mediators was associated with high-virus titers and increased mortality in WNV NY99 infected mice. To our knowledge, this is the first comprehensive study to examine the immunological changes in mice after infection with a naturally non-virulent strain of WNV and will assist to understand the underlying immunopathological mechanisms leading to severe WNV disease.
